# Herpes Zoster in a 13-Year-Old Male Without Prior Varicella Infection

**DOI:** 10.7759/cureus.61155

**Published:** 2024-05-27

**Authors:** Steele L Willoughby, Patrick Burton, James R Carroll

**Affiliations:** 1 Medicine, Edward Via College of Osteopathic Medicine, Spartanburg, USA; 2 Family Medicine, James R. Carroll MD PA, Mullins, USA

**Keywords:** varicella-zoster virus, immunocompetent, pediatric herpes zoster, atypical presentation, herpes zoster virus

## Abstract

Herpes zoster (HZ) typically presents following reactivation of latent varicella-zoster virus (VZV) in adult and geriatric patients with a history of prior varicella infection. Primary VZV infection in patients compliant with vaccine schedules and without any immunocompromising condition is rare, with reactivation leading to HZ being even rarer. This case report details one such example involving a 13-year-old immunocompetent and fully immunized male with HZ despite no history of VZV infection, as well as possible explanatory mechanisms for this uncommon presentation. This case report contributes to a growing body of literature on atypical HZ presentations in pediatric populations without any history of prior VZV infection or exposure.

## Introduction

It is believed that herpes zoster (HZ) is caused by the reactivation of varicella-zoster virus (VZV) or human herpes virus type 3 (HHV 3) located in the posterior nerve root ganglion. Typically, reactivation is associated with age-related immunosenescence, disease-related immunocompromise, or iatrogenic immunosuppression, with age being the major risk factor. Mechanical trauma, physiologic stress, and recent research suggest that SARS-CoV-2 [1, 2] can also play a role in reactivation. As of 2011, "the annual incidence rate (IR) of HZ across all ages was 4.47 per 1,000 person-years (95% confidence interval (CI): 4.44-4.50), which monotonically increased with age from 0.86 (95% CI: 0.84-0.88) for those aged ≤19 to 12.78 (95% CI: 12.49-13.07) for patients ≥80 years. The IR was 8.46 (95% CI: 8.39-8.52) among adults ≥50 years and 10.46 (95% CI: 10.35-10.56) among those aged ≥60 years." [3], indicating that HZ has a highly significant association with advancing age and presents only rarely among pediatric populations.

Vaccination with a live, attenuated VZV strain is the current recommended method of achieving immunity from VZV infection, impeding future HZ eruptions. Primary immunization is achieved by administering a two-dose series at 12 to 15 months of age and four to six years of age [4]. Herpes zoster has been reported to be 78% less common in vaccinated pediatric populations without underlying immunodeficiency when compared to their unvaccinated peers in the United States [5]. Data from other developed countries, however, have indicated a recent increase in HZ rates in vaccinated pediatric populations [6], further supporting the investigation and differential diagnosis of HZ in patients with vaccination and without prior VZV diagnosis.

## Case presentation

A previously healthy, immunocompetent 13-year-old male initially presented to his local urgent care with the chief complaint of left lower extremity myalgias. Following a physical exam and evaluation of his medical history, which included active participation on a basketball team, he was diagnosed with a muscle strain of the left thigh and provided with education on muscle strain symptom management.

Seventy-two hours later, the patient presented to his primary care provider (PCP) with a complaint of a painful rash on his left lower back. The onset of the rash was several hours prior to his presentation to his PCP, and the patient described it as burning and itching. The patient also reported subjective fevers but denied any other associated symptoms. The patient’s parents denied any prior chicken pox diagnosis and affirmed that he had received the varicella vaccine in accordance with routine childhood vaccination scheduling. There is no documented history of immunocompromising diseases (patient or family) or new/altered medication regimens.

**Figure 1:**
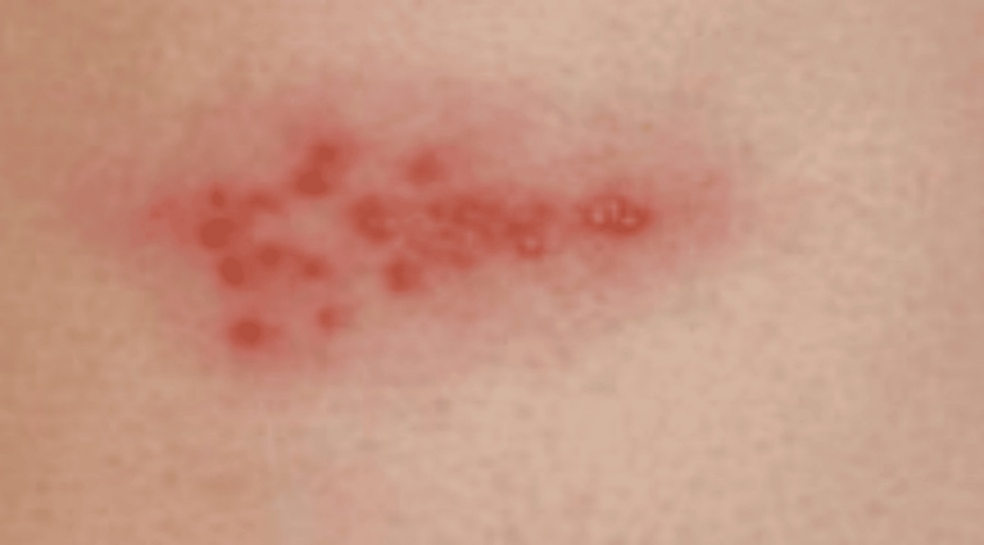
Clinical presentation of the patient after rash eruption

Upon physical examination, a grouped, unilateral vesicular rash with surrounding erythema (Figure 1) was noted in an L4 dermatomal distribution on his left side. The erythematous portion of the rash was blanchable, and there were no other signs indicative of infection. A clinical diagnosis of HZ was made due to the dermatomal distribution of the rash as well as the patient’s clinical history of significant neuropathic pain/myalgia prior to the rash's appearance. The patient was prescribed valacyclovir 1,000 mg three times daily (TID) for seven days, with resolution of his myalgias the day treatment began and resolution of the vesicular rash within five days.

Varicella zoster immunoglobulin M (IgM) and IgG were ordered both on the day of diagnosis and six months following, per parent request (Table 1).

**Table 1:** Patient's laboratory studies Reference interval: negative: <0.91 index; borderline: 0.91−1.09 index; positive: >1.09 index VZV: varicella zoster virus; Ig: immunoglobulin

	Diagnosis	After 6 months
VZV IgM	1.26	<0.91
VZV IgG	>4000	3679

## Discussion

Herpes zoster manifests following the reactivation of the latent VZV. Primary infection with VZV is commonly known as chickenpox, presenting as a wide-spread rash with lesions in different stages of healing and being typically self-limited. Upon reactivation, HZ classically presents as a painful, unilateral vesicular rash in adults, which is currently understood to be due to the age-related decline in virus (VZV)-specific cell-mediated immunity. However, pediatric patients with HZ can present slightly differently, with itching and then subsequent pain, fever, and weakness. Childhood HZ also seems to be slightly more common in males than females, though further data is necessary to confirm this finding [7]. It is thought that both wild-type and vaccine viruses can remain latent to be subsequently reactivated, given the necessity for an explanatory mechanism of disease pathogenesis in cases such as this one [8]. 

Despite the potential for the live, attenuated varicella vaccine to become latent in a small subset of patients, the data in support of childhood vaccination are excellent. Current CDC recommendations for pediatric varicella vaccination are to receive the first dose at age 12 through 15 months and the second dose at age four through six years old [4]. A case-control study published in the Journal of Infectious Diseases found the effectiveness of receiving both vaccine doses to be 98.3% [9], corroborating current CDC recommendations. The prevention of varicella is vital, as post-infection sequelae include Group A streptococcal infections of the skin and soft tissue, pneumonia, encephalitis, cerebellar ataxia, bleeding diatheses, and sepsis [10]. Further, pediatric patients who develop HZ can suffer from debilitating, protracted postherpetic neuralgia.

Clinical diagnosis of HZ can be challenging in atypical presentations such as vaccinated patients, particularly with respect to pediatrics. While data on this topic is sparse given the rarity of pediatric HZ, a previous examination of 39 reported cases found that, among the 33 patients who had received the varicella vaccination, the interval between vaccination and the presentation of HZ symptoms varied from 56 days to approximately nine years, underscoring the necessity of including HZ in the differential diagnosis of any patient with unilateral radiculopathy symptoms, even without the presence of a dermatomal rash [11]. Common disease processes with exanthems similar to HZ include herpes simplex, dermatitis herpetiformis, impetigo, and contact dermatitis presentations. Adequate follow-up is also essential for proper diagnosis and treatment and can further be supported by laboratory studies in cases with atypical presentations. 

Treatment for HZ is dependent on immune status, disease complications, and patient age. Uncomplicated HZ treatment is recommended for any of the following: oral acyclovir 800 mg five times daily for seven days; valacyclovir 1,000 mg TID for seven days; or famciclovir 500 mg three times daily for seven days. Valacyclovir 100 mg TID for seven days has been shown to provide better pain relief than acyclovir 800 mg five times daily for seven days and is likely to have better treatment adherence due to a lower pill burden [12]. When post-herpetic neuralgia is identified, famciclovir has been shown to best reduce neuralgia duration. Regardless of the therapy chosen, pharmacotherapy proves to be more efficacious when implemented within 72 hours of cutaneous involvement.

Complicated HZ, marked by extensive cutaneous eruptions or visceral involvement, requires parenteral support, with acyclovir 10 mg/kg every eight hours for seven to 10 days for those under one year of age and 500 mg/m^2^ for those over one year [13]. Patients with immunocompromised states such as HIV typically require referral to guidelines and infectious disease consults on a case-by-case basis.

## Conclusions

The diagnosis of HZ is infrequent in vaccinated populations, particularly pediatrics. Regardless, it is imperative that clinicians retain a high index of suspicion such that even atypical presentations of HZ can be quickly identified and treated to avoid long-term sequelae. Herpes zoster should be ruled out even in cases of fully vaccinated, immunocompetent pediatric patients who present with unilateral, isolated pruritus or discomfort.
